# Shear bond resistance and enamel surface comparison after the bonding and debonding of ceramic and metallic brackets

**DOI:** 10.1590/2176-9451.19.1.077-085.oar

**Published:** 2014

**Authors:** José Maurício da Rocha, Marco Abdo Gravina, Marcio José da Silva Campos, Cátia Cardoso Abdo Quintão, Carlos Nelson Elias, Robert Willer Farinazzo Vitral

**Affiliations:** 1 MSc in Orthodontics, Adjunct professor, Department of Orthodontics - School of Dentistry - Federal University of Juiz de Fora (FO/UFJF).; 2 PhD in Dentistry, State University of Rio de Janeiro (UERJ). Adjunct professor, Department of Orthodontics, FO/UFJF.; 3 Postdoc in Orthodontics and professor, FO/UFJF.; 4 PhD in Orthodontics and Adjunct professor of Orthodontic, UERJ.; 5 Adjunct professor of Biomaterials, Fluminense Federal University (UFF).; 6 Associate professor, Department of Orthodontics, FO/UFJF.

**Keywords:** Shear bond strength, Tooth enamel, Orthodontic brackets, Scanning electron microscopy

## Abstract

**Objective:**

To evaluate, *in vitro*, the shear bond strength presented by three brands of polycrystalline ceramic brackets and one brand of metallic bracket; verify the adhesive remnant index (ARI) after the tests, and analyze, through scanning electron microscopy (SEM) the enamel surface topography after debonding, detecting the release of mineral particles.

**Methods:**

Sixty bovine lower incisors were used. Three ceramic brackets (Allure^®^, InVu^®^, and Clarity^®^) and one metallic bracket (Geneus^®^) were bonded with Transbond XT^®^. Kruskal-Wallis's test (significance level set at 5%) was applied to the results of share bond and ARI. Mann Whitney's test was performed to compare the pairs of brackets in relation to their ARI. Brown-Forsythe's test (significance level set at 5%) was applied to the results of enamel chemical composition. Comparisons between groups were made with Games-Howell's and the Post-hoc tests.

**Results:**

No statistically significant difference was observed in relation to the shear bond strength loads. Clarity^®^ brackets were the most affected in relation to the surface topography and to the release of mineral particles of enamel (calcium ions).

**Conclusion:**

With regard to the ARI, there was a prevalence of score 4 (40.4%). As for enamel surface topography, the Geneus^®^ bracket was the only one which did not show superficial tissue loss. The InVu^®^ and Clarity^®^ ones showed cohesive fractures in 33.3% and the Allure^®^ in 50%, the latter being the one that presented most fractures during removal.

## Abstract (pt)

**Objetivo:**

avaliar, *in vitro*, a resistência ao cisalhamento apresentada por três marcas de braquetes ortodônticos cerâmicos policristalinos e uma marca de braquete metálico, verificando os índices de resina remanescentes (IRA) após os ensaios, e analisar, por meio de microscopia eletrônica de varredura, as topografias superficiais do esmalte após a descolagem, detectando o desprendimento de partículas minerais desse.

**Métodos:**

foram utilizados 60 incisivos inferiores bovinos. Os braquetes cerâmicos (Allure, InVu e Clarity) e metálico (Geneus) foram colados utilizando-se o adesivo Transbond XT. Para os resultados referentes ao cisalhamento e ao IRA, foi utilizado o teste de Kruskal-Wallis, com nível de significância de 5%. A prova de Mann-Whtiney foi utilizada para comparar os diferentes tipos de braquetes aos pares, quanto ao IRA. Para a análise dos resultados referentes às composições químicas do esmalte, aplicou-se o teste de Brown-Forsythe, com significância de 5%. As comparações entre os grupos foram realizadas utilizando os testes *Post Hoc* e de Games-Hoewell.

**Resultados:**

não foram observadas diferenças significativas quanto às cargas de cisalhamento. O Clarity foi o mais afetado em relação à topografia e ao desprendimento de partículas minerais do esmalte (íons Ca).

**Conclusão:**

em relação ao IRA, foi confirmada a prevalência do escore 4, com um percentual de 40,4%. Em relação à topografia superficial do esmalte, o Geneus foi o único que não apresentou perda de tecido . O InVu e o Clarity apresentaram fraturas coesivas de 33,3% de suas amostras, e o Allure de 50%, sendo o que mais fraturou durante a remoção.

## INTRODUCTION

The need for esthetic orthodontic treatment, especially by adults, culminated in the development of the first ceramic brackets in the late 1980's. Since then, new technologies for manufacturing esthetic brackets have been developed.^10^ Ceramic brackets may be monocrystalline or polycrystalline, according to the manufacturing process. Polycrystalline brackets are produced by precipitation of aluminum oxide particles blended with a binder, and then textured and fired to remove surface imperfections and stress, caused by the cutting process. This manufacturing process may produce structural failures in the accessories. Monocrystalline brackets are produced by a completely different process. Single sapphire crystals involve the combination of aluminum oxide particles at 2,100°C.^13,19^ The crystalline structure of monocrystalline brackets has higher purity than that of polycrystalline ones, with fewer structural failures but higher manufacturing costs.^3^

Bracket bonding became a routine in orthodontic treatment with fixed appliances after Buonocore^4^ introduced the technique of acid-etching the enamel surface. Buonocore^4^ proposed that the enamel surface could be modified by acids in order to become more receptive to bonding. The technique consisted in enamel prophylaxis, in which the enamel is cleaned, dried and freed from saliva, followed by application of acid on its surface. Several materials have been developed for this purpose. Due to presenting adhesive properties,^4^ composite resins have become the main bonding material.

When a bracket is removed, bond failure may occur in the bracket/adhesive interface (adhesive), in the adhesive/enamel interface (adhesive), in the adhesive layer (cohesive), or both (adhesive and cohesive). Failures in the adhesive/enamel interface lead to a higher risk of having enamel fragments removed along with the bracket base resin. Therefore, many authors suggest that the orthodontist use a technique that promotes failure in the bracket/adhesive interface in order to prevent damage to the tooth structure.^2,4,11,12,14,24^

Due to a great difficulty in obtaining extracted human teeth for dental research, a substitute, with similar physical characteristics, has become necessary. Human teeth are morphologically and histologically similar to the teeth of other mammals. Thus, bovine incisors have become a good option in dental research, as they are adequate in size and easily available.^5^

The shear bond test is one of the most simple and widely used tests for determining adhesion resistance of bonded orthodontic brackets.^8^ In this test, the bond is fractured with the application of a force parallel to the adhesive interface. The test may be conducted with a metallic blade or a steel wire loop, as close as possible to the adhesive interface.^8^ Failure starts at the point where the blade applies a normal force, therefore, failure does not always happen at the weakest point.^23^

Scanning electron microscopy (SEM) has a wide range of applications in different fields of knowledge, and provides detailed structural information with a wide magnification range (up to 300,000X). SEM may be coupled to an X-ray energy dispersive spectroscopy (X-EDS) system, which allows the qualitative and semi-quantitative composition of the samples to be determined through emission of characteristic X-rays.^7^

*In vivo* studies may have several variables which can be minimized by *in vitro* research protocols performed with standard procedures and variables as limited as possible, thus yielding more representative and amenable-to-comparison results. The purpose of this study was to perform *in vitro *assessments of the shear resistance of three commercial brands of polycrystalline ceramic orthodontic brackets, using SEM to analyze the superficial enamel topography after bracket debonding and detect the release of mineral particles.

### Proposition

1. Assess, *in vitro*, the shear resistance of three commercial brands of polycrystalline ceramic orthodontic brackets and of one metallic orthodontic bracket, all with mechanical retention.
2. Assess, by means of light microscopy, the adhesive remnant index (ARI) after bracket removal;
3. Assess, with SEM, the enamel superficial topography after bracket debonding.
4. Detect, by means of SEM adapted with an X-EDS microanalysis system, the release of mineral particles from the enamel after removal of ceramic and metallic brackets.
5. Observe and calculate bracket fracture during removal.

## MATERIAL AND METHODS

Sixty recently extracted bovine lower incisors were obtained from the Municipal Abattoir of Juiz de Fora. The inclusion criteria demanded that the teeth had intact buccal surfaces, and no cavities, fractures, stains or enamel lesions.

All teeth were examined under light microscopy with a stereomicroscope (Stemi 2000C, Zeiss), at the Department of Physics of the Federal University of Juiz de Fora. After selection, the teeth were immersed for seven days into a 0.1% thymol-water solution^17^ at room temperature, for asepsis and dehydration prevention. After this period, the remaining soft tissues, calculi and root-adhered bone fragments were removed.^17^

All teeth were subsequently kept at distilled water at 4°C, which was replaced every seven days, for a period not greater than three months before inclusion in the molded samples. Before inclusion, the teeth had their radicular tips sectioned so as to have the same radicular length.^16^ Prior to plaster casting, carboril disk retentions were performed in the roots in order to increase their retention.

Three commercial brands of polycrystalline ceramic orthodontic brackets (Allure /GAC; InVu /TP and Clarity/3M-Unitek) and a metallic orthodontic bracket brand (Geneus/ GAC), all with mechanical retention, were used. They were divided into four groups with 15 brackets each. The study was conducted in two parts: 1) shear test; 2) SEM with X-EDS microanalysis.

To build the molded samples, 26 mm high PVC pipes with a 25 mm internal diameter were used. The molded samples were filled with type IV pink stone plaster^16^ (Vigodent, Bonsucesso, Rio de Janeiro, Brazil). For bracket bonding, the teeth underwent prophylaxis of their buccal surfaces, with pumice stone and water, and were brushed with a Robinson's brush in low-rotation. The teeth were then washed with water for 10 seconds, and dried with an oil-free and humidity-free air spray.

All brackets were bonded with the Transbond XT adhesive (3M Unitek Orthodontics Products, Mowrovia, USA), according to the manufacturer's instructions. The teeth were initially immersed into a 37% phosphoric acid solution (Condac 3M, FGN, Joinvile, Santa Catarina, Brazil) for 15 seconds, sprayed with water for 10 seconds, and dried with an air spray. By the end of the process, they acquired a chalk-white color.

The adhesive (Primer Transbond XT, 3M Unitek Orthodontics Products, Monrovia, USA) was applied and light-cured for 10 seconds.^4^ At last, the brackets were positioned at the center of the buccal surfaces, excess resin was removed and a light-curing process was performed for 20 seconds (ceramic brackets) and 40 seconds (metallic brackets). The Opti Light digital light curing device (Gnatus, São Paulo, Brazil) was used. After bonding, the teeth remained immersed in distilled water at 37ºC, for 24 hours.

In order to standardize the molded samples for the shear test, a 0.021 x 0.025-in rectangular steel wire guide, inserted into the bracket slots and fixed with elastomeric ligatures, was used for positioning the teeth in the PVC pipes. This device allowed the brackets to remain at the same distance from the molded sample bases, and the buccal surfaces of the teeth to lie on a vertical plane parallel to the blade of the testing machine. Thus, all the molded samples were positioned at the base of the testing machine in such a way that the cleaver would be placed between the base and the occlusal tying-wings of the brackets, directing the force to an axis that was parallel to the bonding surface.

The mechanical shear tests were performed in a universal testing machine (EMIC DL 2000) adapted with a microprocessor, at the Post-Graduation Laboratory of the School of Dentistry - Federal University of Juiz de Fora. A 50 Kgf load cell was used at a 0.5 mm/min testing speed. For bracket debonding, the shear load values applied were gradually increased. The data obtained from these tests were stored in a computer directly linked to the mechanical testing device.

In order to analyze the morphology of the enamel debonding surfaces, the secondary electron analysis technique, through SEM (JEOL, JSM5800 LV), was performed at the Military Engineering Institute of Rio de Janeiro. All the images were under magnification of 200X for observation of the entire surface from which photographs were obtained. To detect enamel mineral chemical elements in the debonding surfaces, SEM with X-EDS microanalysis was employed. This test assessed the damage inflicted to the enamel after debonding, being performed in only five teeth of each group (the ones with the smallest ARI).

In addition, two other variables were assessed: 1) ARI after debonding and; 2) frequency of cohesive bracket fractures during the shear tests.

Therefore, all dental elements were analyzed and classified taking into account their ARI^1,6^ after bracket removal. The scores ranged from zero to five, as follows: (0) no resin remained adhered to the tooth after debonding; (1) less than 25% of resin remained adhered to the tooth after debonding; (2) between 25 and 50% of resin remained adhered to the tooth after debonding; (3) between 50 and 75% of resin remained adhered to the tooth after debonding; (4) more than 75% of resin remained adhered to the tooth after debonding and; (5) all resin remained adhered to the tooth after debonding.^1,6^

The results were statistically analyzed and the Shapiro-Wilk's test did not show normal distribution in the sample. Levene's test demonstrated the homoscedasticity of the sample, while Kruskal-Wallis's test was used, with a significance level set at 5%, to obtain the shear test results and calculate the adhesive remnant index (ARI) in the enamel. Furthermore, Mann-Whitney's test, with a significance level set at 1.25% (equivalent to 0.05/4), was used to compare pairs of brackets with regard to their ARI.

To analyze the chemical composition of the enamel (X-EDSD), Brown-Forsythe's test, with a statistical significance of 5%, was used. Comparisons between groups were made with the Post-hoc and Games-Hoewell's tests.

## RESULTS

Table 1 presents the results (in MPa) of the mechanical shear tests. The mean shear resistance values were as follows: GI (9.97 ± 5.29); GII (11.74 ± 4.52), GIII (10.91 ± 4.37); GIV (12.71 ± 5.81). Kruskal-Wallis's test (5% significance) had P = 0.43, showing that no group differed from one another with regard to the central tendency.

**Table 1:** Means, standard deviation, minimum and maximum values and number of teeth regarding the mechanical shear tests (MPa).

Group	Mean ± SD	Minimum	Maximum	Number of teeth
I) Geneus	9.97 ± 5.29	4.05	17.71	15
II) Allure	11.74 ± 4.52	5.72	22.00	15
III) InVu	10.91 ± 4.37	4.93	17.34	15
IV) Clarity	12.71 ± 5.81	6.16	23.22	15
Total	11.33 ± 5.01	4.05	23.22	60

GI (Geneus^®^) was the only group that did not show bracket fracture during the mechanical testing. On the other hand, GII (Allure^®^) had 50% of its brackets with cohesive fractures, while GIII (InVu^®^) and GIV (Clarity^®^) had fractures of five brackets each, corresponding to 33.3% of the sample in each group. Out of the total number of ceramic brackets used (n = 44), 17 fractured, which represents 38.63% of the sample.

As for the ARI results, some teeth could not be analyzed, as the whole bracket base remained adhered to the crown after removal. The results of this analysis are shown in Table 2. Kruskal-Wallis's Test (5% significance) had P = 0.03, showing statistically significant differences between groups with regard to the central tendency.

**Table 2:** Scores for ARI assessment in the study groups.

ARI groups	Scores						Total
	0	1	2	3	4	5	
I) Geneus	0 (0%)	5 (33.3%)	4 (26.7%)	0 (0%)	2 (13.3%)	4 (26.7%)	15
II) Allure	0 (0%)	0 (0%)	1(7.1%)	0 (0%)	9 (64.3%)	4 (28.6%)	14
III) InVu	2 (20%)	1(10%)	2 (20%)	1(10%)	4 (40%)	0 (0%)	10
IV) Clarity	0 (0%)	0 (0%)	0 (0%)	1(7.7%)	6 (46.2%)	6 (46.2%)	13
Total	2	6	7	2	21	14	52

Table 3 shows the differences between pairs with regard to ARI (Mann-Whitney'ss Test). The pairs were analyzed with a general significance level (P = 0.003), and with specific significance levels not greater than 0.0125 (0.05/4). Statistically significant differences were observed between the following brackets: Clarity^®^ and InVu^®^ (P = 0.002); Allure® and InVu^®^ (P = 0.006) and Clarity^®^ and Geneus^®^ (P = 0.002). The other comparisons yielded no statistically significant differences.

**Table 3:** Differences between bracket pairs, according to the ARI.

	Geneus (I)	Allure (II)	InVu (III)	Clarity (IV)
I) Geneus		0.057	0.605	0.002*
II) Allure	0.057		0.006*	0.488
III) InVu	0.605	0.006*		0.002*
IV) Clarity	0.002*	0.488	0.002*	

**Figure 1:**
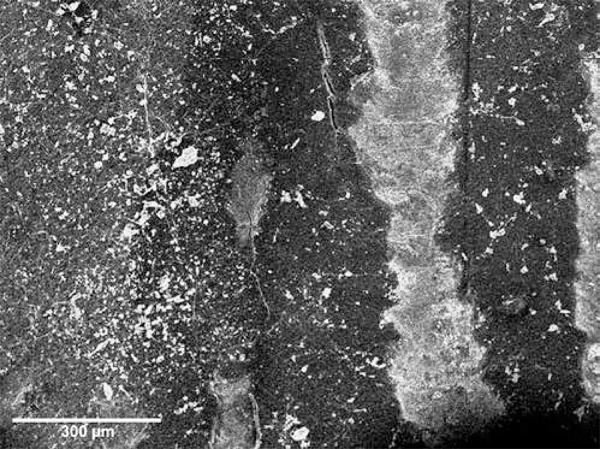
Enamel sample from the Geneus® group, with no superficial tissue loss, showing only small fissures, probably due to the debonding technique.

**Figure 2:**
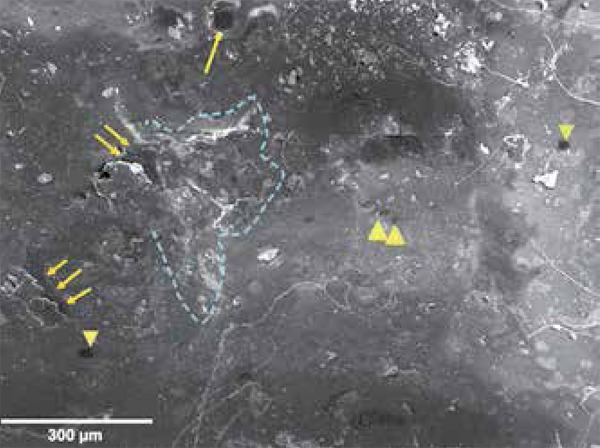
Enamel sample from the Allure® group, showing erosions (yellow arrow) and well-established pores (green arrow point), depressions (orange arrow) and slight loss of the aprismatic enamel layer (blue dotting).

**Figure 3:**
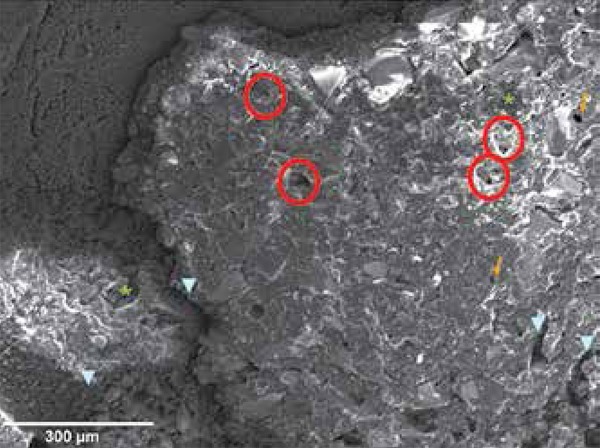
Enamel sample from the InVu® group, showing small craters (green asterisk) surrounded by areas of loss of the aprismatic enamel layer, pores (orange arrow), erosions (red circle) and depressions (blue arrow heads).

**Figure 4:**
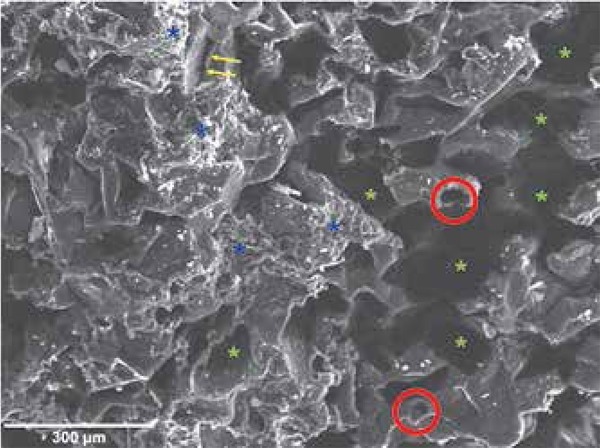
Enamel sample of the Clarity® group, showing significant alterations of the enamel microstructure, with electron-lucent areas, corresponding to craters characteristic of "erosion deepening" (green asterisk), surrounded by extensive loss of the aprismatic enamel layer (blue asterisk) and depressions (yellow arrows) and some erosions (red circle).

Five teeth from each group were selected for enamel analysis with SEM. The choice was based on the molded samples with the smallest ARI value in each group. Since these samples would supposedly have suffered the greatest enamel damage after the shear test, they would probably allow better visualization of the enamel surface. After individual analysis of the 20 teeth (5 from each group) under SEM, all molded samples were found to have microscopic enamel topographic characteristics that were similar within groups. These characteristics are shown in Figures 1 to 4.

All molded samples selected for SEM analysis were also submitted to X-EDS for analysis of the enamel chemical composition.

Table 4 presents the data referring to the chemical composition, described by means and intra-group standard deviations of the percentages of the chemical elements found. Because most chemical elements found were not present in all groups of brackets, statistical analysis was precluded for most of them.

**Table 4:** X-ESD results referring to the mean percentages of calcium present in tooth enamel and their statistical analysis.

Group	Mean	Bracket	Bracket	Mean difference	Mean standard error	p-value
Geneus	52.77	Geneus	Allure	19.10800	13.90868	0.564
			InVu	-7.54000	4.50529	0.421
			Clarity	45.56600 (*)	4.94396	0.000
Allure	33.66	Allure	Geneus	-19.10800	13.90868	0.564
			InVu	-26.64800	13.34571	0.320
			Clarity	26.45800	13.50011	0.327
InVu	60.31	InVu	Geneus	7.54000	4.50529	0.421
			Allure	26.64800	13.34571	0.320
			Clarity	53.10600 (*)	3.01652	0.000
Clarity	7.20	Clarity	Geneus	-45.56600 (*)	4.94396	0.000
			Allure	-26.45800	13.50011	0.327
			InVu	-53.10600 (*)	3.01652	0.000

Only comparative analysis of calcium could be performed, at it was the only chemical element present in all groups of brackets. To this end, Brown-Forsythe's test was used, given that neither Kolmogorov-Smirnov's and Shapiro-Wilk's normality tests nor Levene's equality of variance test found variance normality and similarity between groups.

It is important to point out that, out of all chemical elements found with X-EDS, calcium percentages should be the most noteworthy in the enamel, as higher values would indicate minor damage to the enamel after bracket removal, whereas lower values would suggest severe damage. This is the reason why only the data referring to calcium were presented in this study. The Brown-Forsythe's test reached 5% statistical significance for calcium.

The Post-hoc and Game-Hoewel's tests were used for comparison between groups, with the Clarity^®^ group showing statistically lower percentages of calcium in comparison with the Geneus^®^ and InVu^®^ groups. As for the Allure^®^ group, no statistically significant difference was found between the former and the other groups (Table 4).

## DISCUSSION

Santos et al^17^ and Vicente et al^21^ demonstrated the superiority of Transbond XT^®^ in comparison with glass ionomer cements, with higher bracket adhesive resistance to enamel and higher percentage of fractures in the bracket-adhesive interface. This is the reason why this was the material chosen in this study.

All brackets were conventionally bonded as proposed by Buonocore^4^ in 1955, that is: prophylaxis of the bonding dental surfaces, acid etching, application and light-curing of the Transbond XT^®^ adhesive and bonding with the Transbond XT^®^ resin. Romano^15^ reported the need for acid etching of the enamel to reach shear resistance values that were compatible with clinical use. Savaris and Menezes,^18^ on the other hand, did not observe statistically significant differences in the shear loads between samples in which the Transbond XT adhesive (primer) was light-cured and samples in which no light-curing process was performed. We chose to proceed with acid etching of the enamel and light-curing of the adhesive, as this is a traditional protocol used in shear and enamel surface analysis research.

The mechanical shear tests were performed 24 hours after bracket bonding, with the molded samples being kept in distilled water during this period. We chose this time interval in accordance with Hajrassie and Khier^9^ who did not observe significant differences, *in vitro *or *in vivo*, in the debonding loads of metallic brackets bonded to premolars with Transbond XT^®^ after four different bonding times: 10 minutes, 24 hours, 1 week and 4 weeks.

As for the values obtained with the mechanical shear tests (loads between 9.97 and 12.71 MPa), we concluded that all brackets studied could be successfully used in a clinical scenario, given that the minimum shear resistance loads necessary to tooth movement range from 5.9 to 7.9 MPa, according to Vasques et al.^20^

Cohesive fractures were observed in the three commercial brands of ceramic brackets, with 33.3% for Invu^®^ and Clarity^®^, and 50% for Allure^®^. No fractures were observed in the group of metallic brackets. As for SEM, the samples of the Clarity^®^ group had significant alterations in the enamel microstructure in comparison with the other groups, with electron-lucent areas corresponding to craters, erosion deepening and extensive loss of the aprismatic enamel layer. This was confirmed by X-EDS, in which the Clarity^®^ group had a significantly lower percentage of calcium in the enamel after bracket removal. Likewise, Chen et al^6^ observed cohesive fractures in 25% of the Clarity^®^ brackets after machine-driven debonding. On the other hand, in the same study, Chen et al^6^ observed by means of SEM that most fractures occurred in the bracket/adhesive interface, with no significant enamel damage after bracket removal. However, it is noteworthy that Chen et al^6^ submitted their total sample to SEM with X-EDS microanalysis, whereas in the present study only the teeth with smaller ARI were analyzed, which might have contributed to less favorable results concerning the superficial topography of the enamel.

Savaris and Menezes^18^ as well as Chen et al^6^ did not observe damage or important fractures in the enamel surface after removal of the Clarity^®^ brackets bonded to bovine teeth, reporting that debonding predominantly occurred in the resin layer (cohesive failure, ARI 3), with enamel fracture in just one of the 60 teeth analyzed. Similarly to the study conducted by Chen et al,^6^ the whole sample was submitted to SEM.

Substantial enamel loss and significant enamel damage were observed under SEM analysis for all groups of ceramic brackets. Vilchis, Hotta and Yamamoto^22^ used SEM to observe the superficial enamel topography of premolars submitted to bracket bonding according to two methods: (1) etching with 37% phosphoric acid for 30 seconds + Transbond XT^®^ adhesive + Transbond XT^®^ resin cement; and (2) Transbond Plus Self Etching Primer^®^ (SEP) + Transbond XT^®^ resin cement. Based on the yielded results, they demonstrated that the phosphoric acid etching group had greater enamel loss in comparison to the Transbond Plus Self Etching Primer^®^ group. Additionally, the enamel-adhesive interfaces had more irregularities with phosphoric acid etching. The differences between our findings and some literature reports may be related to such etching, as phosphoric acid was used in our study.

In a search for a more conservative orthodontic bonding, we suggest that further studies comparing Transbond Plus Self Etching Primer^®^, 37% phosphoric acid and other etching materials be undertaken to identify the orthodontic material causing the least enamel damage after bracket removal. This study demonstrated that enamel damage after bracket removal can occur in teeth conditioned with 37% phosphoric acid, with this damage being more frequent and extensive when ceramic brackets are used.

## CONCLUSION

1. There were no statistically significant differences between groups with regard to the shear loads necessary to promote bracket debonding.
2. As for the adhesive remnant index, there were statistically significant differences between the following groups: Clarity^®^ and Invu^®^; Allure^®^ and InVu^®^; Clarity^®^ and Geneus^®^. No statistically significant differences were found between other pairs. In a general context, score 4 prevailed, with 40.4%.
3. Analysis of the enamel superficial topography showed that the Geneus^®^ group was the only one with no superficial tissue loss, having suffered only small fissures, probably due to the debonding technique. All ceramic bracket groups had erosions, pores, depressions and loss of the aprismatic enamel layer, with the Clarity^®^ group being most affected, with significant alterations in enamel microstructure.
4. As for the X-EDSD, the Clarity^®^ group had a significantly lower percentage of calcium in the enamel after bracket removal.
5. Metallic brackets did not fracture during removal. The InVu^®^ and Clarity^®^ groups had fractures in 33.3% of their samples, while the Allure^®^ group had fractures in 50%.
